# Baclofen Inhibits Glioma Proliferation via the MEK/ERK/CREB Pathway

**DOI:** 10.32604/or.2026.079463

**Published:** 2026-05-21

**Authors:** Boqi Zhou, Liping Shen, Xiaojie Lu

**Affiliations:** 1 Wuxi School of Medicine, Jiangnan University, Wuxi, China; 2 Wuxi Neurosurgical Institute, Wuxi No. 2 People’s Hospital (Jiangnan University Medical Center), Wuxi, China; 3 Department of Neurosurgery, Wuxi No. 2 People’s Hospital (Jiangnan University Medical Center), Wuxi, China

**Keywords:** Glioma, baclofen, MEK/ERK/CREB pathway, transcriptome sequencing, cancer neuroscience

## Abstract

**Objectives:** Gamma-aminobutyric acid type B (GABAB) receptors are involved in tumor progression, and baclofen exerts broad-spectrum antitumor effects in various cancers. Nevertheless, its specific function and underlying molecular mechanisms in glioma are still largely unclear. This study aimed to evaluate the effects of baclofen on glioma cells and elucidate the associated signaling pathways. **Methods:** The antitumor effects of baclofen were evaluated in glioma cell lines, and its underlying molecular mechanisms were explored using transcriptome sequencing integrated with Western blotting. The *in vivo* antitumor efficacy of baclofen was further verified in animal models. **Results:** *In vitro* functional assays revealed that baclofen inhibits the proliferation, migration, and invasion of glioma cells in a dose-dependent manner. Transcriptomic sequencing combined with Western blot validation demonstrated that these effects may be mediated by GABAB receptors, leading to suppressed phosphorylation of key molecules in the Mitogen-activated protein kinase kinase (MEK)/Extracellular regulated protein kinases (ERK) pathway, and consequently reduced phosphorylation of the downstream transcription factors cAMP-response element binding protein (CREB) and Fos Proto-Oncogene (FOS). Furthermore, baclofen regulates the epithelial-mesenchymal transition (EMT-like) program. All these effects were abolished by co-treatment with the specific GABAB antagonist CGP35348. *In vivo* experiments using a subcutaneous glioma xenograft model further verified that the continuous use of baclofen in experimental animals also demonstrated certain anti-tumor effects. **Conclusion:** Collectively, these findings demonstrate that baclofen exerts anti-glioma effects through GABAB receptor-mediated inhibition of the MEK/ERK/CREB signaling axis and modulation of the EMT-like pathway, thereby highlighting the potential of baclofen as a therapeutic agent for glioma.

## 1 Introduction

Glioma is the most prevalent primary intracranial tumor [1]. As reported in the CBTRUS Statistical Report, gliomas account for 26.3% of all brain tumors, among which glioblastoma (GBM) represents the most common histopathological subtype of malignant gliomas, accounting for 14.2% of all intracranial tumors [2]. The glioma microenvironment is highly complex, and significant heterogeneity exists across different tumor subtypes, posing substantial challenges to clinical management [3]. Currently, the standard clinical management for glioma patients involves surgical resection, followed by temozolomide-based chemotherapy combined with adjuvant radiotherapy [4], however, the median overall survival of affected patients remains under 15 months [5]. In recent years, with the advancing research on molecular biological alterations associated with glioma progression and microenvironmental regulation, investigating the molecular mechanisms underlying the crosstalk between the nervous system and glioma development, as well as the development of corresponding targeted therapies, has emerged as a research hotspot, with the goal of identifying novel therapeutic targets for glioma.

Emerging evidence indicates that the nervous system is increasingly recognized as a key regulator of tumor progression [6], and the crosstalk among neurons, neurotransmitters, and tumor cells has emerged as a frontier in cancer neuroscience [7]. Meanwhile, numerous preclinical models of malignant tumors have demonstrated that neural activity can modulate tumor initiation and potently drive tumor progression and metastasis. Just as the nervous system regulates tumor progression, tumors can also reshape and hijack the structure and function of the nervous system [8,9]. Accumulating studies suggest that tumor cells can exploit neurotransmitter-initiated signaling pathways to promote uncontrolled proliferation and invasion. Additionally, neurotransmitters can modulate immune cells and endothelial cells within the tumor microenvironment, thereby facilitating tumor progression [10,11].

Gamma-aminobutyric acid (GABA) is the major inhibitory neurotransmitter in the central nervous system (CNS) [12], exerting its biological effects through two primary receptor subtypes: GABAA and GABAB receptors [13]. Baclofen, a synthetic GABA analog, is currently the most specific and potent selective agonist of GABAB receptors in both clinical practice and basic research [14]. Clinically, baclofen is used for treating muscle spasms caused by spinal cord and brain injuries, vertical nystagmus in glioma patients, and intractable hiccups induced by chemotherapy [15,16,17]. The GABAB receptor is a heterodimeric G protein-coupled receptor (GPCR) composed of GABBR1 and GABBR2 subunits, wherein GABBR1 is responsible for binding the endogenous ligand GABA and GABBR2 mediates G protein activation, with the two subunits cooperating to achieve signal transduction [18]. With the advancement of research on the interaction between the tumor microenvironment and the nervous system, the abnormal expression and functional regulatory role of GABAB receptors in various tumor types have been gradually elucidated. GABAB receptors influence tumor progression through multiple processes, including regulating cell proliferation, migration, invasion, epithelial-mesenchymal transition (EMT), and the tumor immune microenvironment, making them potential novel targets for tumor-targeted therapy. Previous studies have detected elevated expression of GABAB receptors in various human cancer cells and tissues, and baclofen has been shown to inhibit tumor growth in rat models [19]. Activation of GABAB receptors can suppress the proliferation of neuroblastoma cells, inhibit the proliferation of lung adenocarcinoma cells, and suppress the growth of cholangiocarcinoma cells [20,21,22]. Furthermore, it can simultaneously suppress cell proliferation and invasion. For example, it inhibits the growth and migration of human hepatocellular carcinoma cells, significantly reduces the proliferative and invasive capacity of colorectal cancer cells, and impedes the proliferation, invasion, and migration of ovarian cancer cells [23,24,25,26]. These findings indicate that GABAB receptor activation exerts antiproliferative effects on a variety of human tumor cells, including pancreatic cancer, lung adenocarcinoma, hepatocellular carcinoma, colon cancer, and breast cancer, with a relatively consistent regulatory pattern across different tumor types [19]. The broad-spectrum antitumor effects of GABAB receptors highlight their great potential for antitumor therapy. Therefore, further exploration and clarification of the underlying mechanisms by which GABAB receptors in the nervous system regulate tumor progression, as well as the development of feasible clinical translation strategies, are essential to provide new approaches for optimizing tumor treatment regimens. 

Therefore, in this study, we aimed to investigate the effects of baclofen on glioma and determine whether baclofen inhibits glioma progression, at least in part, via GABAB receptor-related signaling pathways. 

## 2 Methods and Materials

### 2.1 Cell Culture

Human glioma cell lines, including U251 (Cat. CL-0237), U87 (Cat. CL-0238), T98 (Cat. CL-0583), and LN229 (Cat. CL-0578) were purchased from Pricella (Pricella, Wuhan, China). All cells were cultured in Dulbecco’s modified Eagle’s medium (DMEM, Servicebio, Cat. G4515, Wuhan, China). The medium contains 10% heat-inactivated fetal bovine serum (FBS, Meisen, Cat. CTCC-002-071, Zhejiang, China) and 1% penicillin/streptomycin (Pricella, Cat. PB180120, Wuhan, China). They were incubated at 37°C in an incubator with 5% CO_2_. All cultures were checked for Mycoplasma contamination before experimental use Myco-PCR-Mix (ZQXZBIO, Cat. ZQ505, Shanghai, China) and authenticated using short tandem repeat sequencing (Pricella Biotechnology).

### 2.2 Quantitative Polymerase Chain Reaction (qPCR)

The qPCR assay was performed using a QuantStudio™ 5 Real-Time PCR Instrument (Thermo Fisher Scientific, Cat. A33185, Waltham, MA, USA). The primers were synthesized by Sangon Biotechnology Company (Shanghai, China). According to the manufacturer’s instructions, total RNA was extracted using the FreeZol Reagent kit (Vazyme, Cat. R711, Nanjing, China). In accordance with the manufacturer’s instructions, the process of RNA reverse transcription to cDNA was completed using the HiScript IV cDNA synthesis kit (Vazyme, Cat. R423). Following the manufacturer’s instructions, a 20 μL (the final concentration of the primers: 0.2 μM; the concentration of the template: 500 μM) PCR system consisting of 1.2 μL of cDNA/DNA, 0.4 μL of forward primer, 0.4 μL of reverse primer, 10 μL of ChamQ Universal SYBR qPCR Master Mix (Vazyme, Cat. Q711), and 8 μL of RNase-free dd water was added. In accordance with the manufacturer’s instructions for the PCR program. The gene expression levels were normalized to GAPDH. The PCR protocol commenced with a pre-denaturation step at 95°C for 30 s, followed by 40 amplification cycles consisting of denaturation at 95°C for 10 s and annealing/extension at 60°C for 30 s. Subsequently, a melting curve analysis was performed under the following conditions: 95°C for 15 s, 60°C for 60 s, and a final step at 95°C for 15 s. The reaction system was stored at 37°C. All experiments were independently replicated three times to ensure reproducibility. The primer sequence is: GABBR: 5′-GAGGACGTGAATAGCCGCAG-3′ (forward primer) and 5′-CTGGATCACACTTGCTGTCGT-3′ (reverse primer); GAPDH: 5′-GTCTCCTCTGACTTCAACAGCG-3′ (forward primer); 5′-ACCACCCTGTTGCTGTAGCCAA-3′ (reverse primer).

### 2.3 Cell Viability Assay

The effect of baclofen (MedChemExpress, Cat. No.: HY-B0007, Monmouth Junction, NJ, USA) on cell viability was assessed using the CCK-8 kit (MeilunBio, Cat. MA0218, Dalian, China). Glioma cells were placed in 96-well plates with 1000 cells per well. The experimental group consisted of a blank control group, an untreated control group, and a treated group, each with five replicate wells. After culture, the cells were exposed to multiple drug concentrations (1, 3, 10, 30, 100, 300 μM) for 24 and 48 h. Inject 10 μL of CCK-8 solution into the well containing 100 μL of culture medium, and then incubate at 37°C for 1 h. Optical density measurements were performed at 450 nm (Background absorbance from wells containing culture medium and baclofen reagent without cells was subtracted from all measurements) using a Multiskan FC microplate reader (Thermo Fisher Scientific, Cat. 51119080, Waltham, MA, USA).

### 2.4 Colony Formation Assay

In the clone formation assay, glioma cells in the logarithmic growth stage were implanted into six-well plates, with 1000 cells per well, and each group was repeated four times. After cell attachment (24 h), a new complete medium containing different baclofen drug concentrations (30, 60 μM) was introduced. Cells were maintained at 37°C in a 5% CO_2_ atmosphere for 21 days, and the medium was changed every 3 to 5 days. The obtained clonal colonies were fixed and observed by 0.1% crystal violet (Solarbio, Cat. G1062, Beijing, China) staining, followed by imaging and counting.

### 2.5 Immunofluorescence Staining

Glioma cells in the logarithmic phase of growth were seeded in six-well plates with 50,000 cells per well. After cell attachment (24 h), a new complete medium containing different concentrations of baclofen (30, 60 μM) was introduced. After culturing for 24 h, it was fixed in 4% paraformaldehyde for 30 min, then washed three times with PBS, and permeated with 0.3% TritonX-100 at room temperature for 30 min, then washed three times with PBS, and finally blocked with 5% BSA (Biosharp, Cat. BS114, Beijing, China) for 30 min at room temperature. Subsequently, the cells were incubated overnight at 4°C with anti-Ki-67 antibodies (1:100, Proteintech, Cat. NO. 27309, Wuhan, China). After washing with PBS, apply the secondary antibody (1:5000, BOSTER, Cat. BA1050, Wuhan, China) for 1–2 h at room temperature, and then soak in DAPI (0.5 μg/mL) for 5 min. Finally, place the six-well plate under a fluorescence microscope (ZEISS, microscope model: Axiovert 5 TL FL SCB, Jiangsu, China) for observation (Original magnification:× 400, × 40 objective). The acquired fluorescence images were analyzed for fluorescence intensity using ImageJ software (ImageJ 1.53t, National Institutes of Health, Bethesda, MD, USA).

### 2.6 Wound Healing Assay

Glioma cells in the logarithmic growth phase were seeded (with 50,000 cells per well) into 6-well plates pre-marked on the back. After adherent culture at 37°C with 5% CO_2_ until 90% confluency, straight and clear scratches were made on the cell monolayer using a 200 μL pipette tip. Cells were gently washed 3 times with PBS, then supplemented with serum-free basal medium DMEM (Servicebio, Cat. G4515) containing baclofen at gradients of 0, 30, and 60 μM (four replicates per concentration, with a blank control group). Images were captured immediately under an optical microscope (SITUOLI, Cat. STL-CKXRFA4, Nanjing, China), and additional images were taken at 24, 48, and 72 h post-baclofen treatment. The scratched areas were quantitatively analyzed using ImageJ software 1.53t (National Institutes of Health).

### 2.7 Transwell Invasion Assay

Logarithmic-phase glioma cells were pre-treated with complete medium containing baclofen (0, 30, 60 μM) for 24 h, then digested with trypsin, centrifuged, and resuspended in serum-free basal medium. Matrigel (8.33 mg/mL) was thawed at 4°C one day in advance, diluted 1:8 with pre-cooled serum-free medium, and 100 μL of the diluted Matrigel was added to the upper chamber of Transwell inserts (8 μm pore), followed by incubation at 37°C with 5% CO_2_ for 4 h to allow gelation. The lower chamber of a 24-well plate was filled with 600 μL complete medium, and the Matrigel-coated inserts were placed into the plate. Subsequently, 200 μL of the pre-treated cell suspension (200,000 cells/mL) was added to each upper chamber, and cells were cultured at 37°C with 5% CO_2_ for 24 h. After discarding the medium in the upper chamber, non-migrated or non-invaded cells on the upper surface of the membranes were gently removed with cotton swabs. Inserts were gently washed 3 times with PBS, transferred to a new 24-well plate, and 500 μL 4% paraformaldehyde was added to the lower chamber for fixation at room temperature for 20 min. Following PBS (500 μL) washes, cells were stained with 0.1% crystal violet (Solarbio, Cat. G1062) at room temperature for 10 min, washed again, and the inner surface of the inserts was gently wiped with cotton swabs. After air-drying, images were captured under a microscope (ZEISS, microscope model: Axiovert 5 TL FL SCB). Data were quantitatively analyzed using ImageJ software 1.53t (National Institutes of Health), and each experiment was independently repeated four times.

### 2.8 RNA Sequencing and Gene Enrichment Analysis

U251 cells were cultured in six-well plates with 200,000 cells per well with baclofen (0, 30 μM) for 24 h and dissolved in RNA extraction reagent (Servicebio, Cat. G3013). For both the experimental and control groups, *n* = 3 biologically independent replicates were performed. The RNA integrity numbers (RIN) for the experimental group were 9.4, 9.6, and 9.7, and for the control group were 9.5, 9.6, and 9.4. The corresponding RNA concentrations were 494, 526, and 438 ng/μL for the experimental group, and 498, 444, and 464 ng/μL for the control group. The extracted RNA products were tested by Agilent 4150 TapeStation to confirm the size and integrity of the extracted products. The mRNA was isolated using the Hieff NGS^®^ mRNA Isolation Master Kit V2 (Yeasen Biotechnology, Cat. No. 12629ES96, Shanghai, China), and the subsequent mRNA library was prepared with the Hieff NGS^®^ EvoMax RNA Library Prep Kit (dNTP) (Yeasen Biotechnology, Cat. No. 12341ES97). Library purification was performed with Hieff NGS^®^ DNA Selection Beads magnetic bead purification. The Oligo (dT) magnetic beads were used to enrich mRNA and remove non-coding rRNA. The mRNA was fragmented and reverse transcribed into cDNA using reverse transcriptase. Sequencing linkers were attached to both ends of the cDNA and amplified to form libraries (the insert size was 280 bp; PCR amplification was performed for 11 cycles). Magnetic beads were used to purify the libraries and remove unnecessary impurities. The library was quantified and homogenized, and DNB was prepared after mixing. Load the DNB onto the high-throughput sequencing platform DNBSEQ-T7RS and select PE150 for double-ended sequencing. Genes with |log_2_ Fold Change (FC)| ≥ 1 and *p*-value ≤ 0.05 were defined as differentially expressed genes. The basic transcriptome sequencing data were obtained. Bioinformatics analysis and sequencing were performed by Servicebio (Wuhan).

### 2.9 Western Blot Analysis

The log-growing glioma cells were stored in culture dishes (with 100,000 cells per well) and treated with the drug baclofen for 24 h. Protein extraction was carried out using RIPA dissolution buffer containing 1% protease (Servicebio, Cat. G2008) and 1% phosphatase (Servicebio, Cat. G2007) blocker, followed by centrifugation at 13,440× *g* and separation at 4°C for 15 min to obtain the supernatant. The protein levels were quantified using the BCA assay kit (Servicebio, Cat. G2026), and then 5 × SDS-PAGE protein variation buffer was introduced. The samples were denatured at 100°C for 10 min. The protein samples (25 μg of protein in 5 μL was loaded per lane) were separated by 10% SDS-PAGE and membrane transferred to PVDF and BSA (Biosharp, Cat. BS114) for blocking for 1–2 h. The primary antibodies were incubated overnight at 4°C, followed by a 2-h secondary antibody immersion at room temperature. Anti-rabbit IgG, HRP-linked Antibody (1:5000, Affinity, Cat. S0001, Nanjing, China) and anti-mouse IgG, HRP-linked Antibody (1:5000, Proteintech, Cat. NO. SA00001-1). The primary antibodies used were as follows: GABBR1 (1:2000, Abcam, Cat. AB55051, Shanghai, China), MEK1/2 (1:1000, CST, Cat. 9126, Danvers, MA, USA), P-MEK1/2 (1:2000, CST, Cat. 2338), ERK1/2 (1:1000, CST, Cat. 4695), P-ERK1/2 (1:1000, CST, Cat. 4377), CREB (1:1000, CST, Cat. 4820), P-CREB (1:1000, CST, Cat. 9198), c-FOS (1:1000, CST, Cat. 2250), P-c-FOS (1:1000, CST, Cat. 5348), E-cadherin (1:20,000, Proteintech, Cat. NO. 20874-1-AP), N-cadherin (1:2000, Proteintech, Cat. NO. 22018-1-AP), and Vimentin (1:20,000, Proteintech, Cat. NO. 10366-1-AP), GAPDH (1:5000, Affinity, Cat. T0004). The automated chemiluminescence imaging platform (Tanon-4600, Shanghai, China) enabled protein detection, while the ImageJ software 1.53t (National Institutes of Health) conducted band intensity measurements. The experimental procedure was independently repeated four times.

### 2.10 Tumor Cell Xenograft Nude Mouse Models

A xenograft model involving subcutaneous tumors was implemented using BALB/c nude mice to evaluate the effect of baclofen on glioma *in vivo*. SPF female nude mice (BALB/c nude, total number: 12, weighing 16–18 g, 4–6 weeks old) were from Suzhou SPFbiotech and raised in a specific pathogen-free environment with ad libitum access to food and water. The environment was maintained at a temperature of approximately 22°C, relative humidity 50%, and a 12-h light/dark cycle. All experimental procedures comply with the “Regulations on the Administration of Laboratory Animals”, it was approved by the Animal Ethics Committee of Jiangnan University (Ethics number: JN. No.: 20241030m0640520 [567]). After adaptive feeding for 7 days, to establish an animal model of glioma, U251 cells in 200 μL (approximately 1.0 × 10^7^) were introduced into the lateral abdomen of mice by subcutaneous injection. Seven days later, the mice were randomly divided into two groups (*n* = 6 per group). The sample size was determined based on previous studies and preliminary experiments to ensure adequate statistical power. The treatment group was intraperitoneally injected with baclofen (prepared in sterile PBS) once a day for 3 weeks (20 mg/kg, implanted dose). The control group was intraperitoneally injected with vehicle PBS once a day for 3 weeks. The body weights of nude mice were recorded every 7 days. In addition, we measured the tumor volume once a week. The calculation formula for tumor volume is as follows: Volume = 1/2 × length × width^2^. During the experiment, animal health status was monitored daily, and no animals died or were excluded before the endpoint. At the end of the experiment, these mice were euthanized by cervical dislocation under anesthesia. The tumor was removed and weighed.

### 2.11 Data Analysis of Databases

GEO (https://www.ncbi.nlm.nih.gov/geo/) provides a single-cell sequencing database of human glioma cells, and uses the internal data of GSE134269 for search and analysis. The dataset SCDS0000041 and the single-cell Data with sample number SCSP0000480 were processed and analyzed through the Cell-omics Data Coordinate Platform (CDCP). Single-cell RNA-seq data analysis was performed using the CDCP proprietary platform (Official website: https://db.cngb.org/cdcp/). The analytical pipeline of the platform was strictly followed, including (data normalization, feature selection, dimension reduction, and clustering analysis) in accordance with the platform’s official documentation [27]. The survival analysis of GABBR1 gene in glioma patients was performed by GEPIA2 database. Subsequently, survival analysis was performed to validate the prognostic significance of GABBR1 in glioma patients using the Chinese Glioma Genome Atlas (CGGA) public database.

### 2.12 Statistical Analysis

Experimental data were statistically analyzed using GraphPad Prism version 10.0 (GraphPad Software, San Diego, CA, USA). All experiments were independently repeated at least 3–5 times to ensure result reliability. Quantitative data are presented as the mean ± standard deviation (SD) to describe the central tendency and dispersion of the datasets. The two groups were compared using an unpaired *t*-test. Differences among multiple groups were assessed using one-way analysis of variance (ordinary one-way ANOVA). Statistical significance was determined by a two-tailed *p*-value < 0.05, with hierarchical notation based on the degree of difference: *p < 0.05 (statistically significant), **p < 0.01 (highly significant), ***p < 0.005 (extremely highly significant), ****p < 0.0001 (profoundly significant), and ns (no significant difference between groups).

## 3 Results

### 3.1 Expression of GABAB Receptor GABBR1 Gene in Glioma

**Figure 1:**
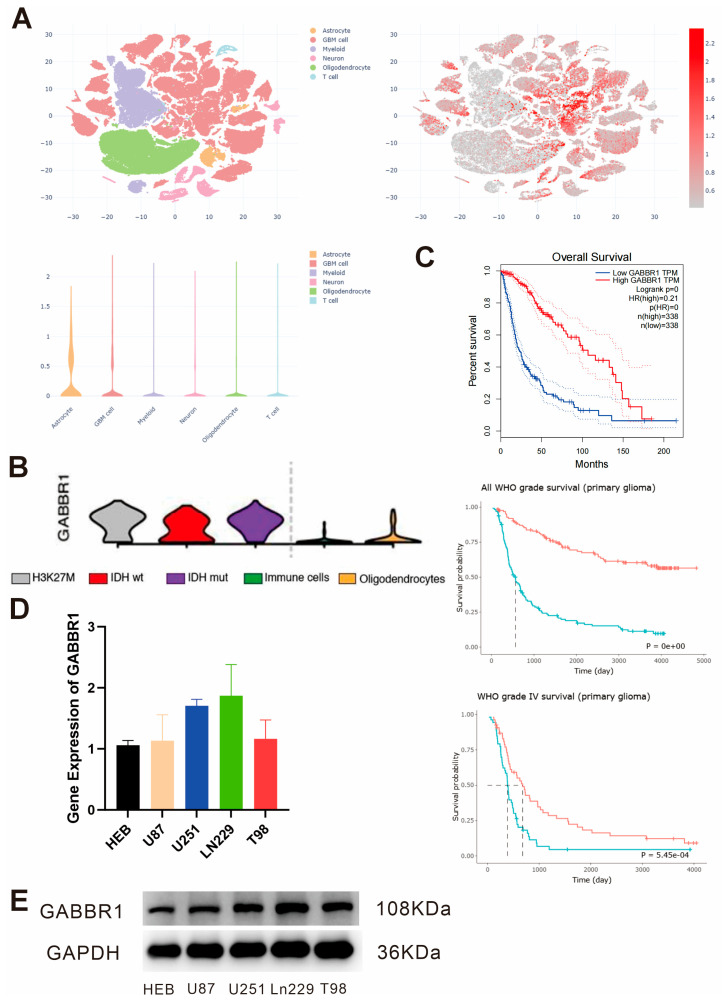
The expression of Gamma-aminobutyric acid B receptors (GABAB) receptor genes on gliomas. (**A**) Single-cell clustering analysis of glioma tissues and relative GABBR1 expression from the CNGB database. (**B**) Relative expression of GABBR1 in different cell types from the GEO database. (**C**) Survival curves of glioma patients with low and high GABBR1 expression from the GEPIA database and further validated in the CGGA public database. (**D**) Relative GABBR1 expression in glioma cells and normal human brain glial cells, *n* = 3. (**E**) Western blot (WB) analysis of GABBR1 protein expression in glioma cells and normal human brain glial cells.

To clarify the expression pattern of GABAB receptors in glioma cells, analysis of the single-cell CNGB database revealed that GABBR1 is predominantly expressed in glioma cell populations and astrocytes (Fig. 1A). Subsequent analysis of single-cell data from the GEO database showed high GABBR1 expression in H3K27M-mutant diffuse midline glioma, IDH-mutant diffuse astrocytoma, and IDH-wild-type glioblastoma (Fig. 1B). Survival analysis of GABBR1 expression using the GEPIA database generated survival curves for glioma patients, indicating that the overall survival of patients in the high GABBR1 expression group was significantly longer than that in the low expression group. Prognostic validation was further performed using the CGGA public database to confirm the survival significance of GABBR1 (Fig. 1C). Furthermore, quantitative real-time polymerase chain reaction (qPCR) and Western blot (WB) analyses were performed to detect GABBR1 expression in glioma cells and normal glial cells, confirming high GABBR1 expression in glioma cells (Fig. 1D,E). Consistent with the single-cell database results, these findings demonstrate abundant expression of GABAB receptors in glioma cells, and the GABBR1 gene may serve as a prognostic factor for glioma, suggesting that GABAB receptors are involved in the development and progression of glioma.

### 3.2 Effect of Baclofen on Glioma Cell Proliferation

**Figure 2:**
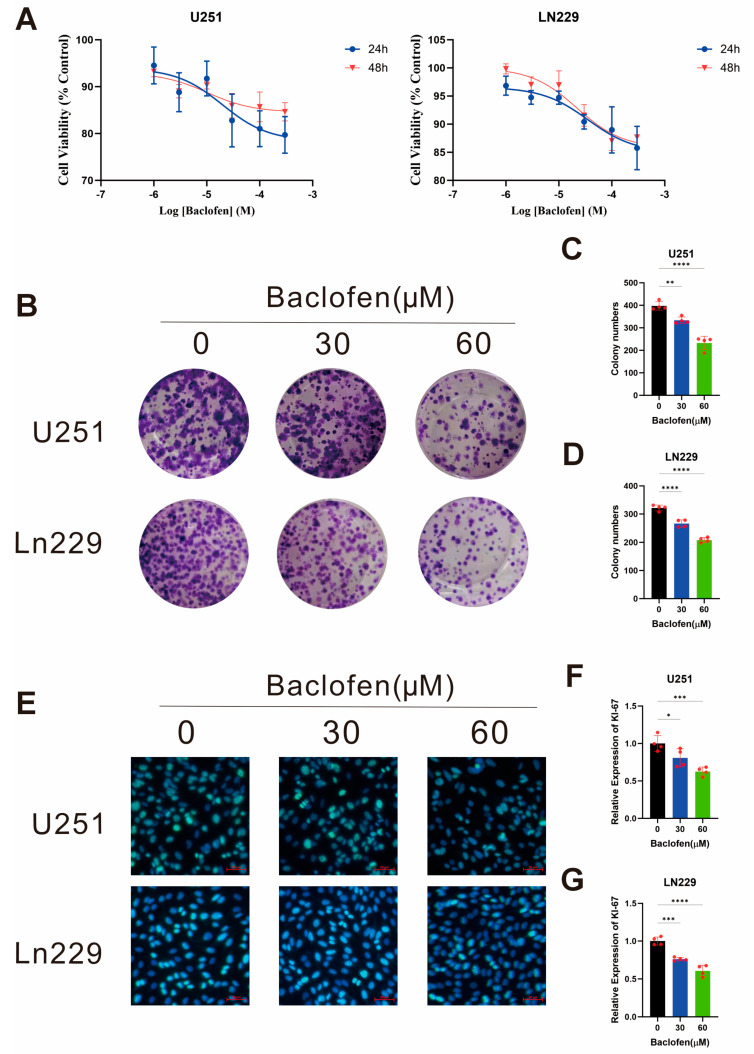
The effect of baclofen on the proliferation of glioma cells. (**A**) Viability of U251 and LN229 glioma cells treated with baclofen as detected by CCK-8 assay, *n* = 5. (**B**) Effect of baclofen on the colony formation of U251 and LN229 glioma cells. (**C**) Quantitative statistical plots of colony formation results of U251 glioma cell, *n* = 4. (**D**) Quantitative statistical plots of colony formation results of U229 glioma cell, *n* = 4. (**E**) Effect of baclofen on the Ki-67 proliferation index in U251 and LN229 glioma cells. Scale bar: 100 μm. Original magnification: ×400 (×40 objective). (**F**) Statistical plots of immunofluorescence intensity for proliferation indices in baclofen-treated U251 glioma cells, *n* = 4. (**G**) Statistical plots of immunofluorescence intensity for proliferation indices in baclofen-treated U229 glioma cells, *n* = 4. (*p < 0.05, **p < 0.01, ***p < 0.001, ****p < 0.0001, all indicate comparison with the control group).

To determine the effect of baclofen on glioma cell proliferation, U251 and LN229 glioma cell lines with relatively high GABBR1 expression were selected for proliferation assays. *In vitro* interventions were conducted using baclofen at different concentrations (0–300 μM) on U251 and LN229 cells. The results showed a significant concentration-dependent inhibitory effect of baclofen on the proliferation of both glioma cell lines, whereas the treatment duration had no significant impact on the inhibitory efficacy (Fig. 2A). After 24 h of baclofen treatment, the half-maximal inhibitory concentration (IC50) values for U251 and LN229 cells were calculated as 33.36 μM and 35.41 μM, respectively. Given the consistent antiproliferative effect of baclofen on glioma cells, 30 μM was selected as the standard concentration for subsequent experiments, with a treatment duration of 24 h. To further verify the antiproliferative effect of baclofen, colony formation assays and Ki-67 immunofluorescence staining were performed using baclofen at concentrations of 0 μM, 30 μM, and 60 μM. At 30 μM baclofen, the colony-forming capacity of both U251 and LN229 cells was reduced, and this inhibitory effect was significantly enhanced at 60 μM (Fig. 2B–D). Similarly, the Ki-67 proliferation index was inhibited at 30 μM baclofen and markedly decreased at 60 μM (Fig. 2E–G). These results confirm that baclofen not only exerts short-term inhibitory effects on glioma cell growth and reduces proliferation indices but also effectively impairs long-term colony-forming capacity. Collectively, these data demonstrate that baclofen can effectively inhibit the proliferative activity of glioma cells *in vitro*, with the inhibitory effect significantly strengthening with increasing drug concentration.

### 3.3 Effect of Baclofen on Glioma Cell Migration and Invasion

**Figure 3:**
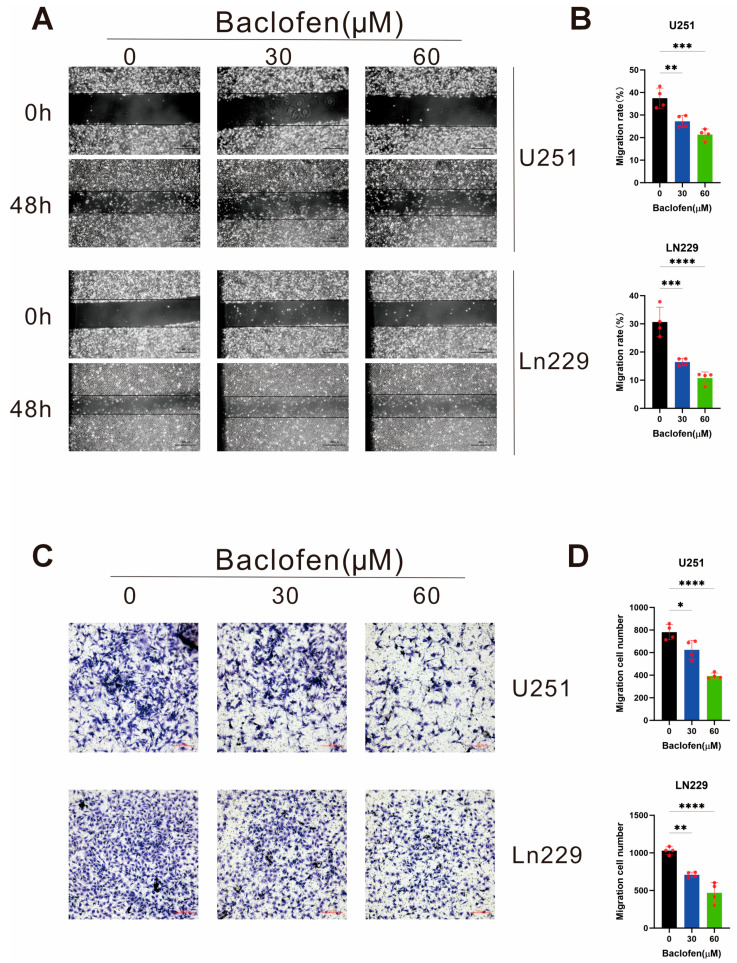
The effect of baclofen on the migration and invasion of glioma cells. (**A**) Migration of U251 and LN229 glioma cells treated with baclofen as detected by wound-healing assay. Scale bar: 500 μm. Original magnification: ×40 (×4 objective). (**B**) Statistical plot of migration capacity in baclofen-treated glioma cells from wound-healing assays, *n* = 4. (**C**) Invasive capacity of U251 and LN229 glioma cells treated with baclofen as determined by Transwell invasion assay. Scale bar: 200 μm. Original magnification: ×100 (×10 objective). (**D**) Statistical plot of invasive capacity in baclofen-treated glioma cells from Transwell invasion assays, *n* = 4. (*p < 0.05, **p < 0.01, ***p < 0.001, ****p < 0.0001, all indicate comparison with the control group).

To further explore the effect of baclofen on glioma cell migration and invasion, wound-healing assays were performed to evaluate migration capacity, and Transwell invasion assays were used to assess invasive potential. Wound-healing assays with baclofen at concentrations of 0 μM, 30 μM, and 60 μM showed a concentration-dependent inhibitory effect of baclofen on glioma cell migration (Fig. 3A,B). Migration capacity was reduced at 30 μM baclofen and significantly inhibited at 60 μM. Transwell invasion assays with the same concentration gradient revealed a similar concentration-dependent inhibitory effect on glioma cell invasion (Fig. 3C,D). Invasive capacity was significantly suppressed at 30 μM baclofen, with a more pronounced effect at 60 μM. These results confirm that baclofen can inhibit the migration and invasion of glioma cells, with the inhibitory effect significantly enhanced with increasing drug concentration.

### 3.4 Transcriptome Sequencing of Glioma Cells Treated with Baclofen

**Figure 4:**
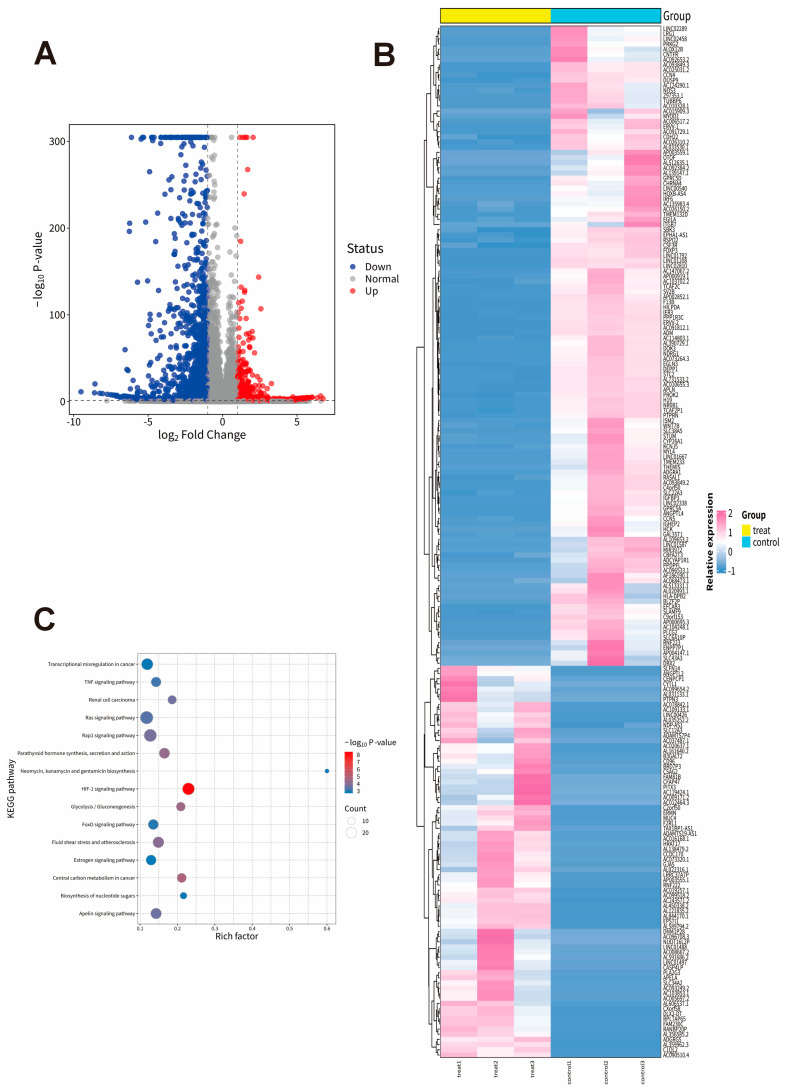
Differential gene expression and pathway enrichment analysis in baclofen treated glioma cells. U251 cells were treated with baclofen for 24 h, and RNA was collected for eukaryotic reference-based transcriptome sequencing. DEGs were analyzed to generate volcano plots (**A**) and differential expression heatmaps (**B**), where upregulated genes are shown in red, downregulated genes in blue, and non-regulated genes in gray (|log_2_ Fold Change (FC)| ≥ 1, *p*-value ≤ 0.05). KEGG pathway enrichment analysis of baclofen-treated U251 glioma cells (**C**).

To elucidate the signaling pathway mechanisms underlying the inhibitory effect of baclofen on glioma cells, U251 cells were treated with baclofen at concentrations of 0 μM and 30 μM. Total RNA was extracted and subjected to eukaryotic reference-based transcriptome sequencing. Differentially expressed genes (DEGs) were identified with thresholds of |log_2_ Fold Change (FC)| ≥ 1 and *p*-value ≤ 0.05. A total of 1402 downregulated genes and 750 upregulated genes were detected, with volcano plots and heatmaps of DEGs shown in Fig. 4A,B. KEGG pathway enrichment analysis of baclofen-treated glioma cells revealed significant enrichment in pathways such as transcriptional misregulation in cancer, TNF signaling pathway, Ras signaling pathway, Rap1 signaling pathway, and HIF-1 signaling pathway, and so on (Fig. 4C). These results provide insights into the specific mechanism by which baclofen inhibits the proliferation and growth of glioma cells.

### 3.5 Effect of Baclofen on the MEK/ERK Molecular Signaling Pathway in Glioma Cells

**Figure 5:**
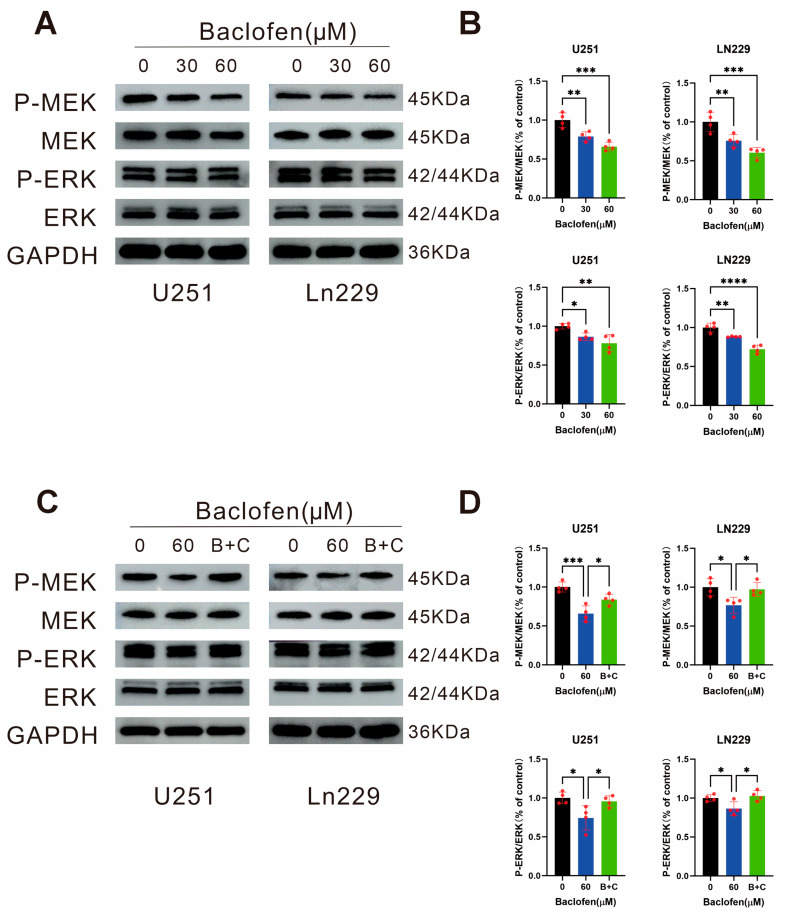
The effect of baclofen on the Mitogen-activated protein kinase kinase (MEK)/Extracellular regulated protein kinases (ERK) pathway in glioma cells. (**A**) WB assay detecting the expression and phosphorylation levels of the MEK/ERK pathway in glioma cells treated with different concentrations of baclofen for 24 h. (**B**) Statistical analysis of the expression and phosphorylation levels of the MEK/ERK pathway in glioma cells treated with baclofen for 24 h, *n* = 4. (**C**) WB assay detecting the expression and phosphorylation levels of the MEK/ERK pathway in glioma cells treated with baclofen alone or in combination with CGP35348 (B + C) for 24 h. (**D**) Statistical analysis of the expression and phosphorylation levels of the MEK/ERK pathway in glioma cells treated with baclofen alone or in combination with CGP35348 (B + C) for 24 h, *n* = 4. (*p < 0.05, **p < 0.01, ***p < 0.001, ****p < 0.0001, all indicate comparison with the control group).

To clarify the molecular mechanism of baclofen’s inhibitory effect on glioma cells, WB assays were performed to determine the expression and phosphorylation levels of key proteins after 24-h treatment with baclofen at concentrations of 0 μM, 30 μM, and 60 μM. Based on the transcriptome sequencing data, DEG changes, and related pathway analysis, WB assays were used to verify the expression and phosphorylation levels of MEK1/2 and ERK1/2 proteins. The results showed that baclofen treatment significantly reduced the phosphorylation levels of MEK1/2 and ERK1/2 in glioma cells, indicating that baclofen may inhibit the phosphorylation-activated effect of the MEK/ERK pathway, thereby blocking downstream signal transduction associated with proliferation and growth (Fig. 5A,B). This result is consistent with previous findings in pancreatic cancer cells, suggesting that baclofen exerts antitumor effects through the ERK pathway in tumor cells. To further confirm whether the regulation of MEK/ERK pathway phosphorylation levels is induced by baclofen-activated GABAB receptors, thereby inhibiting proliferation, CGP35348, a specific selective GABAB receptor antagonist, was co-administered to antagonize the effects of GABAB receptors. Subsequent detection of MEK1/2 and ERK1/2 expression and phosphorylation levels showed that the baclofen-induced reduction in MEK1/2 and ERK1/2 phosphorylation was changed by CGP35348 (B + C), which represents the combination of baclofen and CGP35348 (Fig. 5C,D). These findings indicate that baclofen may be mediated by GABAB receptors on glioma cells, leading to decreased phosphorylation levels of proteins in the MEK/ERK signaling pathway, and inhibits glioma cell proliferation by downregulating the phosphorylation levels of key pathway factors, confirming that baclofen is associated with GABAB receptors are involved in the regulation of tumor cell proliferation. Collectively, these results suggest that baclofen may exert its antitumor effects by activating GABAB receptors to inhibit the phosphorylation of the MEK/ERK pathway.

### 3.6 Effect of Baclofen on Key Transcriptional and Regulatory Factors in Glioma Cells

**Figure 6:**
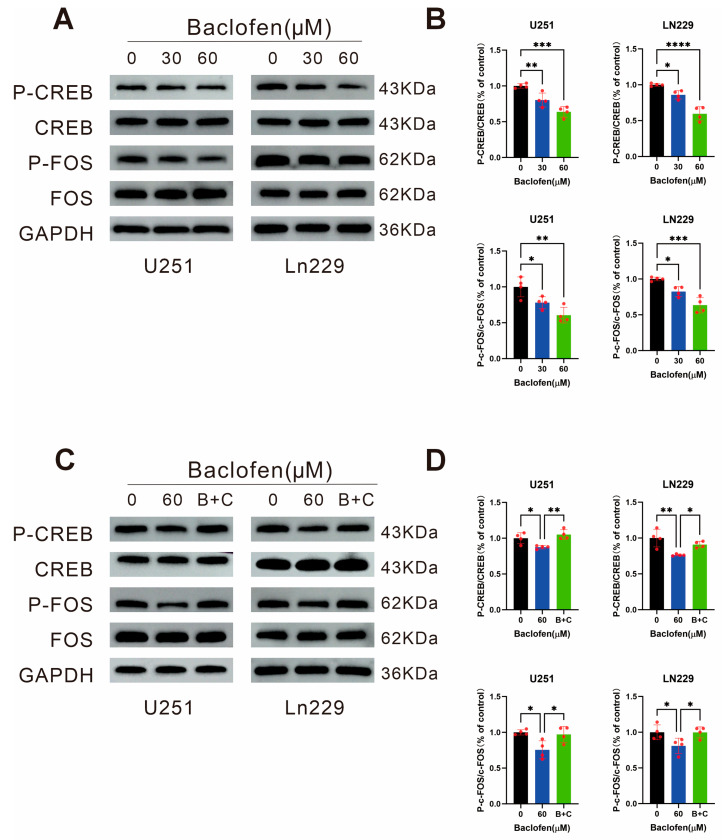
The effect of baclofen on transcription factors of glioma cells. (**A**) WB assay detecting the expression and phosphorylation levels of key transcriptional regulatory factors CREB and c-FOS in glioma cells treated with different concentrations of baclofen for 24 h. (**B**) Statistical analysis of the expression and phosphorylation levels of CREB and c-FOS in glioma cells treated with baclofen for 24 h, *n* = 4. (**C**) WB assay detecting the expression and phosphorylation levels of CREB and c-FOS in glioma cells treated with baclofen alone or in combination with CGP35348 (B + C) for 24 h. (**D**) Statistical analysis of the expression and phosphorylation levels of CREB and c-FOS in glioma cells treated with baclofen alone or in combination with CGP35348 (B + C) for 24 h, *n* = 4. (*p < 0.05, **p < 0.01, ***p < 0.001, ****p < 0.0001, all indicate comparison with the control group).

To further clarify the molecular mechanism changes induced by the MEK/ERK pathway upon baclofen in glioma cells, WB assays were performed to verify the effects on key pathway-related factors after 24-h treatment with baclofen at concentrations of 0 μM, 30 μM, and 60 μM. The results showed that baclofen treatment significantly reduced the phosphorylation levels of the key transcriptional regulatory factor cyclic AMP response element-binding protein (CREB) and the proto-oncogene c-FOS (Fig. 6A,B). These findings indicate that baclofen may downregulate the phosphorylation level of CREB, leading to decreased phosphorylation of c-FOS protein and ultimately exerting antitumor effects. To confirm whether these phosphorylation changes are induced by baclofen, which is associated with GABAB receptors, CGP35348 was co-administered to antagonize GABAB receptors. The results showed that CGP35348 changed the baclofen-induced decrease in CREB and c-FOS phosphorylation levels. (B + C) represents the combination of baclofen and CGP35348 (Fig. 6C,D). This indicates that GABAB receptor-mediated transcriptional misregulation in cancer occurs in glioma, and baclofen can downregulate the phosphorylation level of the key transcriptional regulatory factor CREB, which may be mediated by activating GABAB receptors, ultimately reducing c-FOS protein phosphorylation and inhibiting glioma cell proliferation.

### 3.7 Effect of Baclofen on the EMT-Like Pathway and Its Key Proteins in Glioma Cells

**Figure 7:**
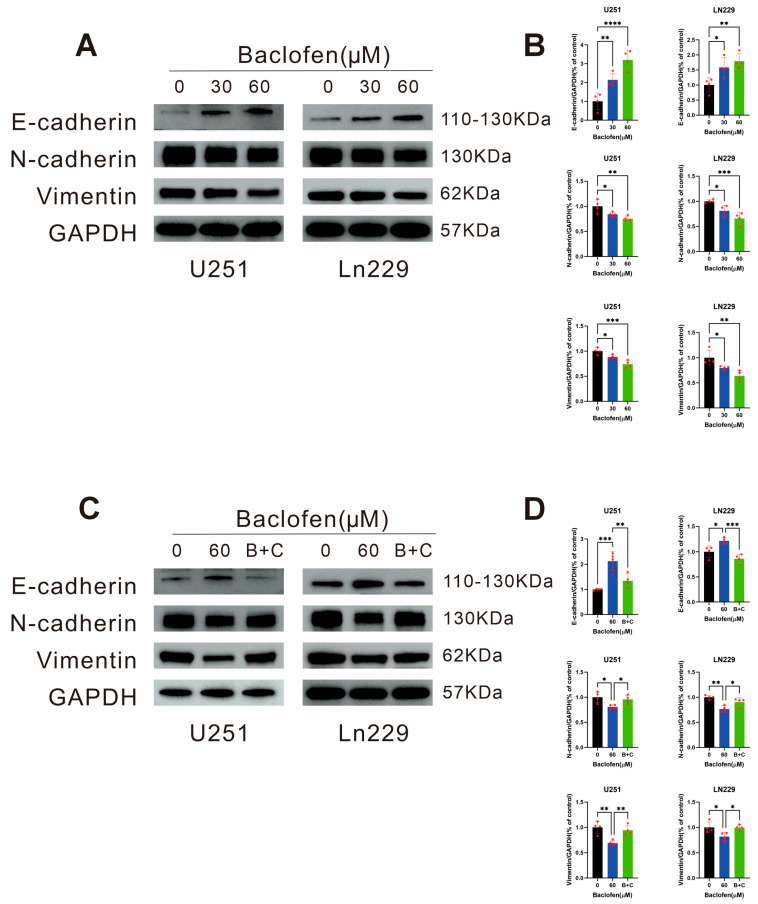
The effect of baclofen on the proteins related to the EMT-like pathway in glioma cells. (**A**) WB analysis was conducted to examine the expression levels of (E-cadherin, N-cadherin, and Vimentin) key proteins in the EMT pathway in glioma cells following 24-h treatment with baclofen at varying concentrations. (**B**) Statistical analysis was performed to quantify the expression levels of E-cadherin, N-cadherin, and Vimentin in glioma cells after 24-h exposure to baclofen, *n* = 4. (**C**) WB assay was employed to detect the expression patterns of E-cadherin, N-cadherin, and Vimentin in the EMT pathway of glioma cells treated with baclofen alone or in combination with the GABAB receptor antagonist CGP35348 (B + C) for 24 h. (**D**) Statistical analysis was carried out to assess the expression levels of E-cadherin, N-cadherin, and Vimentin in glioma cells following 24-h treatment with baclofen monotherapy or baclofen combined with CGP35348 (B + C), *n* = 4. (*p < 0.05, **p < 0.01, ***p < 0.001, ****p < 0.0001, all indicate comparison with the control group).

To explore whether the inhibitory effect of baclofen on glioma cell migration and invasion is associated with the EMT-like pathway, WB assays were performed to verify the effects on pathway-related factors after 24-h treatment with baclofen at concentrations of 0 μM, 30 μM, and 60 μM. The results showed that baclofen treatment upregulated the expression of E-cadherin and downregulated the expression levels of N-cadherin and Vimentin, indicating that baclofen modulates the EMT-like pathway to inhibit glioma cell migration and invasion (Fig. 7A,B). To confirm whether the inhibition of migration and invasion is mediated by GABAB receptors, co-administration of CGP35348 to antagonize GABAB receptors reversed the changes in EMT-like-related proteins. (B + C) represents the combination of baclofen and CGP35348 (Fig. 7C,D). These results fully demonstrate that baclofen may be mediated by GABAB receptors on glioma cells and exerts antitumor effects by inhibiting migration and invasion through the EMT-like pathway.

### 3.8 Preliminary Anti-Tumor Effect of Continuous Baclofen In Vivo

**Figure 8:**
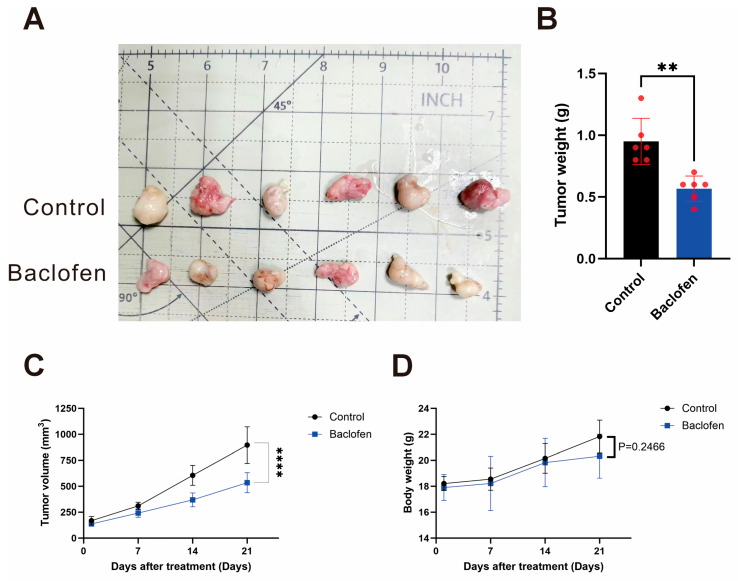
The effect of baclofen on the growth of glioma in nude mice. (**A**) Representative images of tumor size comparison after complete dissection of the subcutaneous glioma xenograft model in nude mice. (**B**) Statistical analysis of tumor weight in the two groups, *n* = 6. (**C**) Dynamic changes in tumor volume continuously monitored after the initiation of administration in the subcutaneous glioma xenograft model in nude mice, *n* = 6. (**D**) No significant difference in mouse body weight was observed after continuous baclofen treatment, *n* = 6. (**p < 0.01, ****p < 0.0001, all indicate comparison with the control group).

To investigate whether baclofen inhibits glioma growth *in vivo*, a subcutaneous glioma xenograft model was established to systematically evaluate the effect of baclofen administration on glioma progression. Seven days after tumor formation, mice were divided into two groups (*n* = 6/group): a control group receiving a placebo and a treatment group receiving baclofen. The results showed that the tumor size and weight in the baclofen treatment group were significantly smaller and lighter than those in the control group, indicating that baclofen can inhibit glioma growth *in vivo* and suggesting its potential *in vivo* antitumor effects (Fig. 8A,B). Tumor volume and mouse body weight were monitored daily after the initiation of administration. The results showed no significant difference in body weight changes between the treatment and control groups, while tumor volume increased rapidly in the control group and remained significantly smaller in the baclofen treatment group throughout the study period (Fig. 8C,D). A preliminary anti-tumor effect of continuous baclofen administration was also seen *in vivo*.

## 4 Discussion

As the most common malignant tumor of the central nervous system, glioma is characterized by high invasiveness, poor prognosis, and limited treatment options, highlighting the urgent need to explore novel therapeutic targets and strategies [28]. In recent years, research in the field of cancer neuroscience has revealed that neurotransmitters and their receptors play critical roles in tumor development and progression, providing a new direction for tumor treatment [29]. Gamma-aminobutyric acid (GABA), the major inhibitory neurotransmitter in the central nervous system, has been shown to regulate various tumors through its receptor-mediated signaling pathways [30]. Baclofen, a classic selective agonist of GABAB receptors [31,32], has been previously confirmed to inhibit the proliferation of multiple tumor cells, including pancreatic cancer, lung adenocarcinoma, and colon cancer cells, by activating GABAB receptors [19]. However, its role and specific regulatory mechanism in glioma remain unclear. Based on this, the present study systematically investigated the effects of baclofen on glioma, aiming to provide a new theoretical basis and potential targets for glioma treatment.

Glioma development is closely associated with the dysregulation of signal transduction pathways, primarily the abnormal activation of tyrosine kinase receptor pathways, including the PI3K/AKT and RAS/MAPK/ERK signaling pathways, which promote glioma cell growth and angiogenesis [33]. The PI3K/AKT/mTOR pathway is one of the most frequently mutated pathways in glioblastoma patients [34]. Studies have confirmed that the combined activation of the Ras and AKT pathways can induce glioblastoma formation in mice [35]. Additionally, the Wnt/β-catenin signaling pathway exerts oncogenic activity in glioma proliferation, apoptosis inhibition, and invasion, potentially through the regulation of multiple genes via microRNAs (miRNAs), such as miR-21 targeting TCF4, miR-27b targeting c-myc and cyclin D1, and miR-23b directly targeting VHL [36,37,38]. Multiple factors related to the Wnt/β-catenin signaling pathway are associated with glioma progression, migration, and poor prognosis in glioblastoma patients [39]. Meanwhile, the TP53/MDM2/CDKN2A pathway plays a key role in tumor suppression, but this pathway is dysregulated in most glioblastoma patients [40]. GABAB is functionally coupled to Gi/o proteins, which can modulate downstream intracellular signaling cascades, including the MAPK/ERK pathway, through multiple intermediate regulators [41]. The RAS/MAPK/ERK signaling pathway is upregulated in glioma and regulates tumor cell proliferation and invasion [42], providing a basis for antitumor research targeting this pathway.

The core mechanism by which baclofen exerts its antitumor effects may involve the activation of GABAB receptors, thereby directly inhibiting cell proliferation. Studies have shown that GABA recruits β-arrestins through GABAB receptors to promote the activation of the JNK cascade, ultimately inducing apoptosis in various tumor cells and inhibiting their proliferation [43]. Additionally, researchers have found that baclofen can activate GABAB receptors to inhibit GSK-3β activation, thereby suppressing NF-κB function and ultimately inhibiting colorectal cancer cell proliferation [44]. In breast cancer cells, baclofen can significantly inhibit cell growth, migration, and invasion by downregulating flavin expression, upregulating β-catenin expression, and regulating EMT markers [45]. Furthermore, in pancreatic cancer cells, GABA or baclofen can block isoproterenol-induced ERK1/2 activation in a GABAB receptor expression-dependent manner, directly affecting tumor cell proliferation [46]. These cross-tumor studies suggest that the antitumor effects of baclofen may be conserved across different tumor types. In the present study, we found that baclofen exerts antitumor effects on glioma cells by concentration-dependently inhibiting the phosphorylation of the MEK/ERK signaling pathway, thereby downregulating the phosphorylation levels of the downstream transcription factor CREB and its target protein c-FOS to inhibit proliferation. Meanwhile, we demonstrated that baclofen can also modulate glioma cell invasion and migration through the EMT-like pathway. Because gliomas are not tumors of epithelial origin, the altered ability to invade and migrate may be caused by a mesenchymal-like transition. These findings are consistent with the regulatory effects of baclofen on other tumors in cross-tumor studies, further confirming the potential universal tumor-suppressive role of baclofen across different tumor types, as well as the role of the MEK/ERK signaling pathway in tumor growth, providing new insights and experimental basis for targeted glioma therapy.

This study initially confirmed the antitumor effects of baclofen on glioma, including inhibiting the phosphorylation of the MEK/ERK pathway, regulating the phosphorylation levels of downstream transcription factors, suppressing glioma cell proliferation, inhibiting migration and invasion through the EMT-like pathway, and exerting inhibitory effects *in vivo*. However, this study has several limitations that need to be addressed in future research. Notably, the concentrations of baclofen used in the present study (10–100 μM) are consistent with those widely applied in previous tumor-related studies. Although these concentrations may exceed physiological levels, several lines of evidence suggest that the observed anti-proliferative effects are unlikely to represent non-specific cytotoxicity or off-target effects. First, baclofen is a well-characterized and selective agonist for GABAB receptors, and its biological actions are primarily mediated through this receptor. Second, the inhibitory effect of baclofen on cell viability exhibited a typical sigmoidal dose-response relationship and reached a clear plateau, such that further increases in concentration did not enhance its anti-tumor effect. This pattern is consistent with a receptor-mediated mechanism rather than non-specific cytotoxicity. Nevertheless, given that the present study did not include genetic approaches such as GABAB receptor knockout or knockdown to formally validate receptor specificity, we are cautious in interpreting the underlying mechanism. Accordingly, the anti-tumor effects of baclofen observed in this study are considered to potentially involve GABAB receptor-dependent pathways, rather than being definitively ascribed to this receptor. First, at the molecular mechanism level, although the MEK/ERK/CREB/c-FOS pathway has been identified as an important pathway through which baclofen regulates glioma cell proliferation, the signaling events associated with baclofen and GABAB receptors remain unclear. Therefore, further pathway inhibition or activation experiments are needed to explore whether Gi/o proteins or other intermediate signaling molecules are involved, so as to establish the causal relationship. Second, the conclusions are drawn based on observations in U251 and LN229 cells and did not investigate differences in baclofen sensitivity among different molecular subtypes of glioma cells (H3K27M-mutant diffuse midline glioma, IDH-mutant diffuse astrocytoma, and IDH-wild-type glioblastoma). As an FDA-approved clinically used drug, baclofen possesses a well-characterized safety profile and favorable pharmacokinetic properties. Baclofen has been documented to cross the blood–brain barrier, supporting its potential to target intracranial glioma. The present study used a subcutaneous xenograft model, which cannot fully mimic the native glioma microenvironment, particularly interactions with neurons, glia, and the blood–brain barrier. Future studies using orthotopic models will be required to further confirm these findings. However, potential CNS-related side effects, including drowsiness, dizziness, and muscle weakness, should be carefully considered in future clinical applications. Several translational challenges remain, including optimizing delivery strategies to enhance local concentration within the tumor microenvironment, minimizing off-target central effects, and validating efficacy in clinically relevant orthotopic glioma models.

Further studies are warranted to fully evaluate the safety, tolerability, and therapeutic potential of baclofen as a repurposed agent for glioma treatment. Additionally, the subcutaneous xenograft model used in this study to explore the *in vivo* antitumor effects of baclofen cannot fully simulate the physiological tumor microenvironment of glioma (interactions with nerve cells, glial cells, and immune cells in the central nervous system) or reflect the developmental characteristics of glioma within the nervous system. Therefore, the anti-tumor effects observed in the subcutaneous model should be considered preliminary evidence, rather than definitive support for therapeutic efficacy against glioma in a physiological setting. These issues are crucial for in-depth glioma research and require focused attention in subsequent studies. Based on the limitations of this study, future research directions may focus on the following aspects: 1) Further elucidate the signaling networks associated with baclofen and GABAB receptors, and to better clarify the role of baclofen in glioma, genetic approaches will be used to knockdown or knockout the GABAB receptor gene, to clarify its exact correlation with baclofen. Meanwhile, the molecular mechanism underlying the interaction between GABAB receptors and the MEK/ERK pathway will be explored. We will further screen and validate the key target genes of CREB/c-FOS that regulate cell proliferation, with the aim of refining the molecular mechanism network of baclofen against glioma; 2) Establish an orthotopic glioma xenograft model combined with GABAB receptor gene modification models to further verify the *in vivo* antitumor effects of baclofen and the core role of GABAB receptors; 3) Validate the antitumor effects of baclofen and the correlation between GABAB receptor expression and prognosis in different molecular subtypes of glioma cells and clinical samples, providing a basis for the precise clinical application of baclofen.

## 5 Conclusion

Glioma, an invasive CNS malignancy with poor prognosis and limited treatments, requires novel targets. This study explores baclofen, consistent with cross-tumor studies, baclofen inhibits glioma via dose-dependent MEK/ERK suppression, reduced CREB/c-FOS phosphorylation, and EMT-like modulation. The mechanism underlying the effects of baclofen is speculated to involve GABAB receptors. Despite its efficacy, limitations exist (unclear GABAB signaling, untested subtype sensitivity, poor subcutaneous models), with future research focusing on clarifying signaling, using orthotopic models, and clinical validation for precise therapy.

## Data Availability

The data that support the findings of this study are available from the corresponding author upon reasonable request. All relevant data are within the paper.
